# *Solanum linnaeanum* and *Solanum sisymbriifolium* as a sustainable strategy for the management of *Meloidogyne chitwoodi*

**DOI:** 10.1038/s41598-020-77905-2

**Published:** 2021-02-10

**Authors:** Laura Soraia Perpétuo, Maria J. M. da Cunha, Maria Teresa Batista, Isabel Luci Conceição

**Affiliations:** 1 Centre for Functional Ecology-Science for People and the Planet (CFE), Department of Life Sciences, University of Coimbra, 3000-456 Coimbra, Portugal; 2 Polytechnic Institute of Coimbra, High School of Agriculture, Department of Agronomic Sciences and Research Centre for Natural Resources, Environment and Society (CERNAS), Bencanta, 3045-601 Coimbra, Portugal; 3 Chemical Process Engineering and Forest Products Research Centre (CIEPQPF), Faculty of Pharmacy of the University of Coimbra, Pólo das Ciências da Saúde, Azinhaga de Santa Comba, University of Coimbra, 3000-548 Coimbra, Portugal

**Keywords:** Plant sciences, Zoology

## Abstract

Root-knot nematodes (RKN), *Meloidogyne* spp., are important crop pests that cause severe losses in crop production worldwide, reducing both productivity and crop quality. *Meloidogyne chitwoodi* Golden, O'Bannon, Santo & Finley, 1980 is considered a quarantine organism by the European and Mediterranean Plant Protection Organization (EPPO) causing damage in tomato and potato crops. The development of nonchemical and sustainable management strategies to reduce nematode damage is crucial. The resistance of *Solanum linnaeanum* Hepper & P.-M.L. Jaeger and *S. sisymbriifolium* Lamarck cv. Sis 6001 to *M. chitwoodi* was evaluated based on gall index (GI), the Bridge & Page (1980) rating chart and reproduction factor (RF). Both plant species were resistant to *M. chitwoodi*. *Solanum linnaeanum* had an average of 519 small root swellings/plant, with 45% adult nematodes inside the roots, all males. *Solanum sisymbriifolium* had GI ≤ 2 and RF ≤ 1 with a high percentage (69%) of nematodes inside the roots that did not develop beyond the sexually undifferentiated second-stage. The use of *S. linnaeanum* as a new source of resistance is a good alternative for the control of RKN in the quest to develop nonchemical and sustainable management strategies to protect crops.

## Introduction

Plant-parasitic nematodes (PPN) are a focus of intense scientific research because they represent an important constraint for global food production, reducing yields and crop quality worldwide^1–4^. Most agricultural fields are infested with at least one PPN species and annual crop losses caused by them are estimated to be between 8.8 and 14.6% of total World crop production^5,6^. The nematodes with the greatest economic impact are the root-knot nematodes (RKN), *Meloidogyne* spp. Many species of this genus are quite aggressive, and they are widely distributed across a range of climatic conditions^3,7,8^. Some species are considered quarantine pests by the European and Mediterranean Plant Protection Organization (EPPO) and interfere with international trade^9^.

In Europe, RKN are increasingly important and they are responsible for significant yield losses, reaching up to 100% in tomato, *S. lycopersicum* (L.) H. Karst, one of the major vegetable crops cultivated and consumed internationally^10,11^.

Potato, *S. tuberosum* L., is an important staple crop in Portugal and, according to “Instituto Nacional de Estatística” (INE), an average of 431,686 ha of potatoes are produced every year^12^. *Meloidogyne chitwoodi* Golden, O'Bannon, Santo & Finley, 1980 is considered a quarantine organism by EPPO (EPPO A2 list: No. 227) causing damage and making tubers unsuitable for consumption or processing. Even though *M. chitwoodi* is present in most major potato producing areas, its detection in tubers can lead to the rejection of entire shipments. Since the 1990s it has become prevalent in vegetable and potato production areas^13,14^.

The global distribution and wide range of hosts of RKN explains the high economic impact of these nematodes and the difficulties found in their management^11^. Although RKN are impossible to eradicate, it is crucial to reduce the amount of damage they cause if productivity is to be sustainable^5^. Their control is mainly achieved by the cultural practices of crop rotation and the use of resistant cultivars, combined with nematicide application^15,16^. Despite the fact that the use of chemical pesticides is an effective control strategy, this is an expensive method and European legislation (Directive 69/465/CEE; Directive 2009/128/EC) is very strict regarding the use of nematicides in the field, focusing mainly on environmental safety issues and health risks. The increase of environmental concerns and regulatory restrictions on the application of chemical products in conventional systems have meant that the use of plant resistance to control PPN has increased. The most effective, environmentally friendly, and economical means of controlling *Meloidogyne* spp. is the use of resistant cultivars^10^. However, up to now, there are no potato cultivars resistant to RKN^9^.

Therefore, other control measures are being developed, including the use of plants as trap crops as an alternative to chemical pesticides^17–20^. A trap crop is a plant species attractive to pests from another crop but in which the pest fails to survive or reproduce. Many wild *Solanum* species display resistance to *Meloidogyne* spp., although the resistance is often not complete, and the nematodes may form a few galls or eggs. A potentially useful source of resistance occurs in *S. sisymbriifolium* Lamarck^21,22^.

*Solanum sisymbriifolium,* originates from warm, temperate South America, is an annual or perennial erect, rhizomatous, shrubby weed with an extensive root system and spiny leaves, currently distributed throughout the world and invasive in some countries. Interest in the study of this plant has increased since it was proved to be a good trap crop against potato cyst nematodes (PCN), *Globodera* spp. It was introduced in The Netherlands following research that identified the species as the most suitable candidate among a diversity of species tested as potential trap crops^17,20,22–25^. Several studies have been done with this plant and it has proved to have effects on hatching, mortality, infectivity and/or reproduction in several nematode species, including PCN, RKN and root-lesion nematodes (RLN)^18,20,23,24,26–28^. The effects of *S. sisymbriifolium* on nematodes depend on the cultivars used, the genus and species of nematode present and on biotic and abiotic conditions^10^.

In Portugal, *S. sisymbriifolium* is not part of the native flora^29^, but other plants of the same genus, for instance *S. linnaeanum* Hepper & P.-M.L. Jaeger, which is similar to *S. sisymbriifolium* (Supplementary Fig. S1), are present in the South of Portugal^30,31^. It is an invasive species of plant, spiny, with an extensive root system, and is probably native from Southern Africa although it is a common weed in North Africa and Southern Europe^30,32^.

*Solanum sisymbriifolium* is also known to be a source of resistance, or partial resistance, to some diseases and plant pests, including fungi, bacteria, nematodes and insects. *Solanum linnaeanum* has been much less studied but has potential as a source of resistance to some fungi and viruses^33–35^.

The main goals of the present study were: to evaluate the potential value of two wild *Solanum* plants, *S. linnaeanum* and *S. sisymbriifolium*, in controlling agricultural pests such as RKN; to assist in the development of sustainable and ecofriendly management methods of key enemies in agriculture, in order to reduce dependence on chemical pesticides; and to improve crop productivity. The resistance shown by *S. linnaeanum* and *S. sisymbriifolium* cv. Sis 6001 was evaluated against *M. chitwoodi*. Although some studies have been made with *S. sisymbriifolium* and PPN and it is already used as a trap crop in some places, not much is known about *S. linnaeanum* and its effects on nematodes. New sources of resistance may result in the development of sustainable and nonchemical management strategies to protect crops against PPN. This is the first report of *S. linnaeanum* being used in Portugal for the management of PPN.

## Results

**Figure 1:**
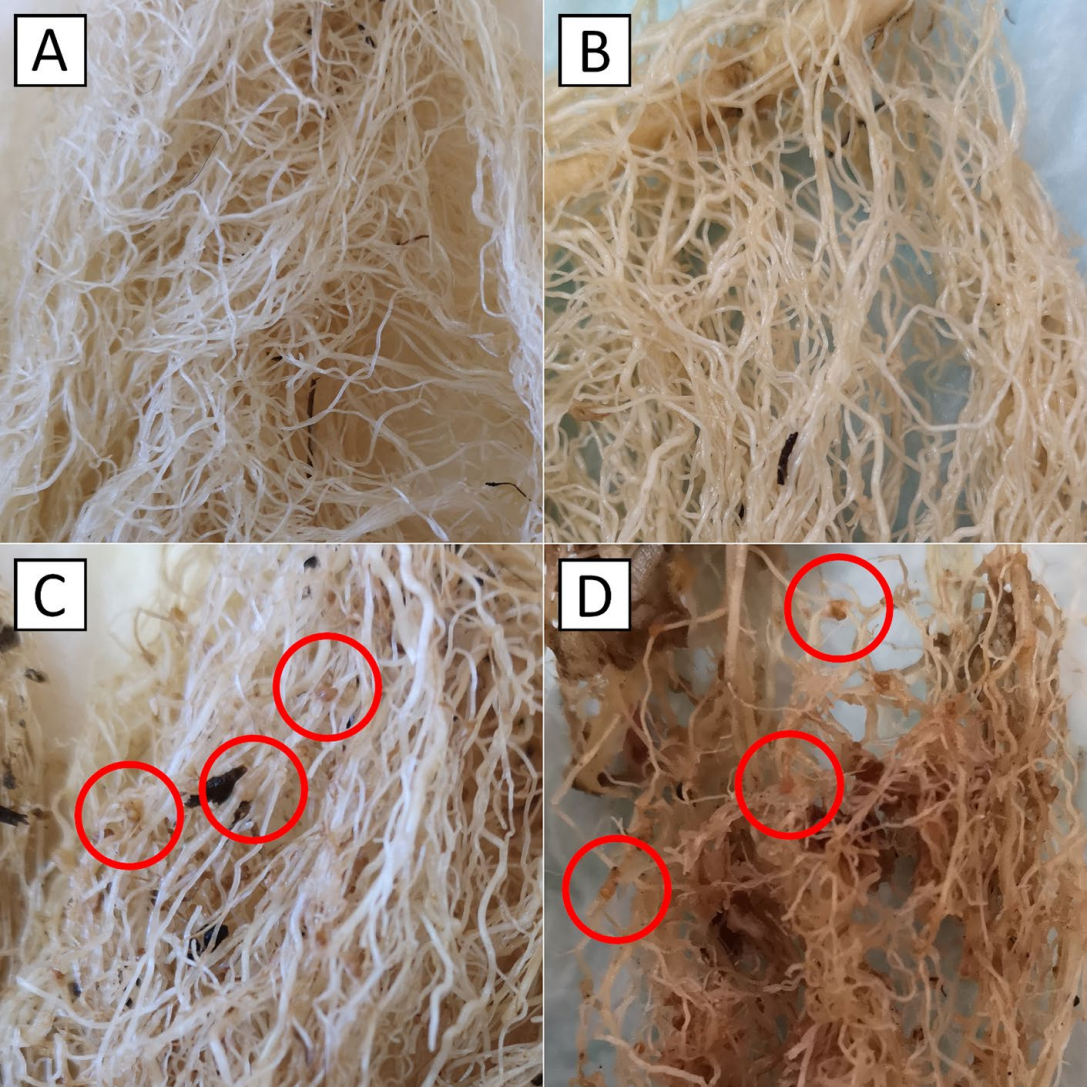
*Solanum linnaeanum* (**A**)*, S. sisymbriifolium* cv. Sis 6001 (**B**), *S. lycopersicum* cv. Coração de boi (**C**) and *S. tuberosum* ssp. *tuberosum* cv. Désirée (**D**) roots. Galls with egg masses marked in controls (**C**, **D**) with red circles.

The environmental conditions used, and the period of the experiments, proved to be appropriate and sufficient for the development and reproduction of the nematodes. The results showed that there was no reproduction of *M. chitwoodi* in *S. linnaeanum* and that there was only a little reproduction in *S. sisymbriifolium* cv. Sis 6001, when compared with the reproduction in the susceptible plants used as controls, tomato cv. Coração de boi and potato cv. Désirée (Fig. 1 and Supplementary Fig. S1).

Soil sterility was confirmed by the absence of galls and egg masses in uninoculated plants. The values of GI = 5 and RF > 1 (Table 1) obtained in the controls, tomato and potato, confirmed the viability of the inocula and that environmental conditions were favorable for penetration, development and reproduction of *M. chitwoodi*.

**Table 1:** Resistance degree (RD) of *Solanum linnaeanum, S. sisymbriifolium* cv. Sis 6001 and respective controls (tomato and potato), 70 days after inoculation with 5000 eggs of *Meloidogyne chitwoodi* per plant (averages of ten replicates).

*Solanum* species and cultivars	GI	RF	RD
*S. linnaeanum*	0*	0*	R
*S. sisymbriifolium* cv. Sis 6001	0*	0*	R
*S. lycopersicum* cv. Coração de boi^a^	5**	9**	S
*S. tuberosum* ssp. *tuberosum* cv. Désirée^a^	5**	10**	S

GI (Gall Index) on a scale of 0–5; RF (Reproduction Factor) = Pf/Pi where Pf = final population and Pi = initial population; Resistance degree (RD): R = resistant (GI ≤ 2 and RF ≤ 1); S = Susceptible (GI > 2 and RF > 1); ^a^Controls. Data from five plants per treatment of two independent experiments (n = 10) were submitted to ANOVA, and comparison of means by LSD test (P < 0.05) was carried out for GI and RF. Values with the same symbol are not significantly different.

The same was verified by the index in the Bridge & Page (1980) rating chart^36^, which varied between 4 in tomato plants, 40% roots infested with larger knots but main roots clean, and 5 in potato plants, 50% roots infested with knotting on parts of main roots. All inoculated plants presented symptoms of yellowing, wilting and leaf drop, related to the presence of nematodes.

**Figure 2:**
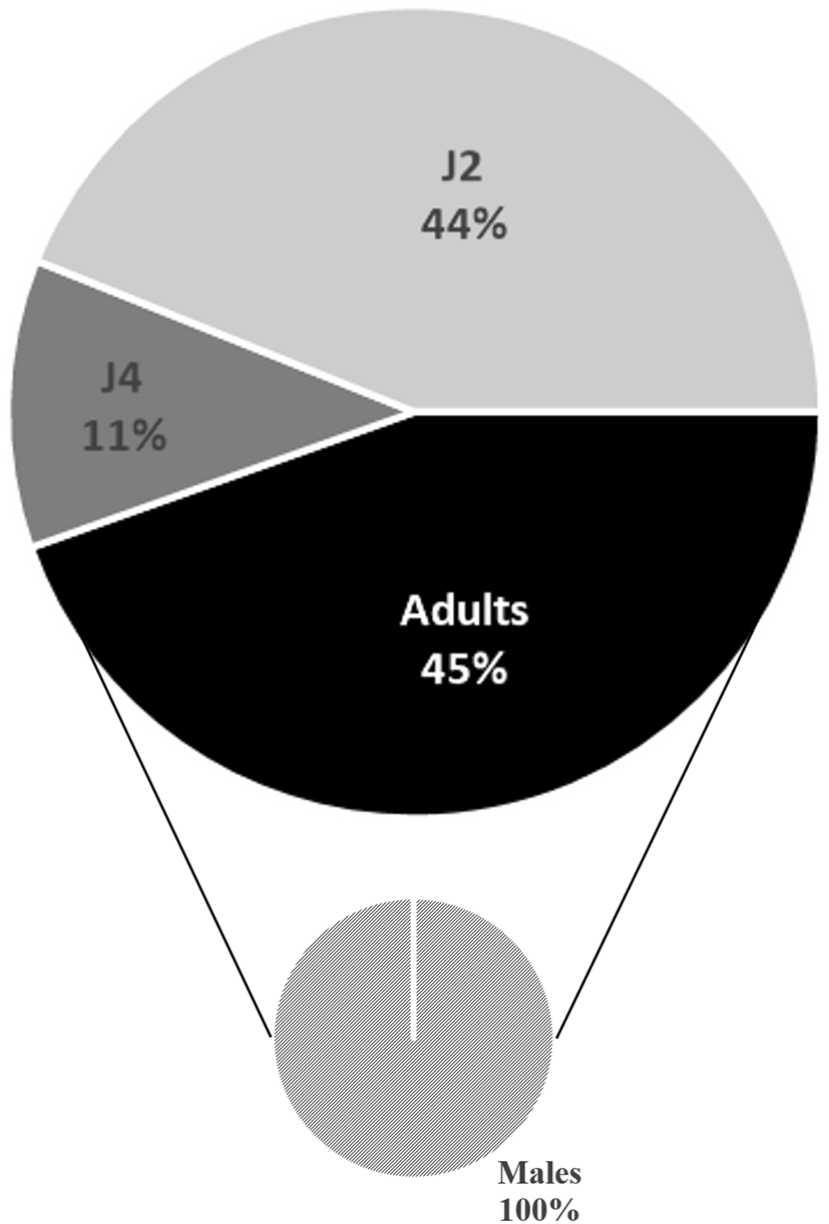
Number of nematodes in different development stages, J_2_, J_4_ and adults (all males), on *Solanum linnaeanum* root systems. The percentages are averages of the two assays (5 replicates/assay).

*Solanum linnaeanum* was considered resistant to *M. chitwoodi* (Table 1 and Supplementary Table S1). The index in the Bridge & Page (1980) rating chart^36^ was 0, because no egg masses developed on the roots. Observation of the stained root systems showed the presence of many small root swellings (average of the 10 replicates was 519 small root swellings/plant) and nematodes inside the roots (total numbers values between 329 and 883 per plant) (Supplementary Table S2). Almost all the nematodes inside the roots were second-stage juveniles (J_2_) (44%) or adults (45%) and all the adults were males (100%) (Fig. 2).

**Figure 3:**
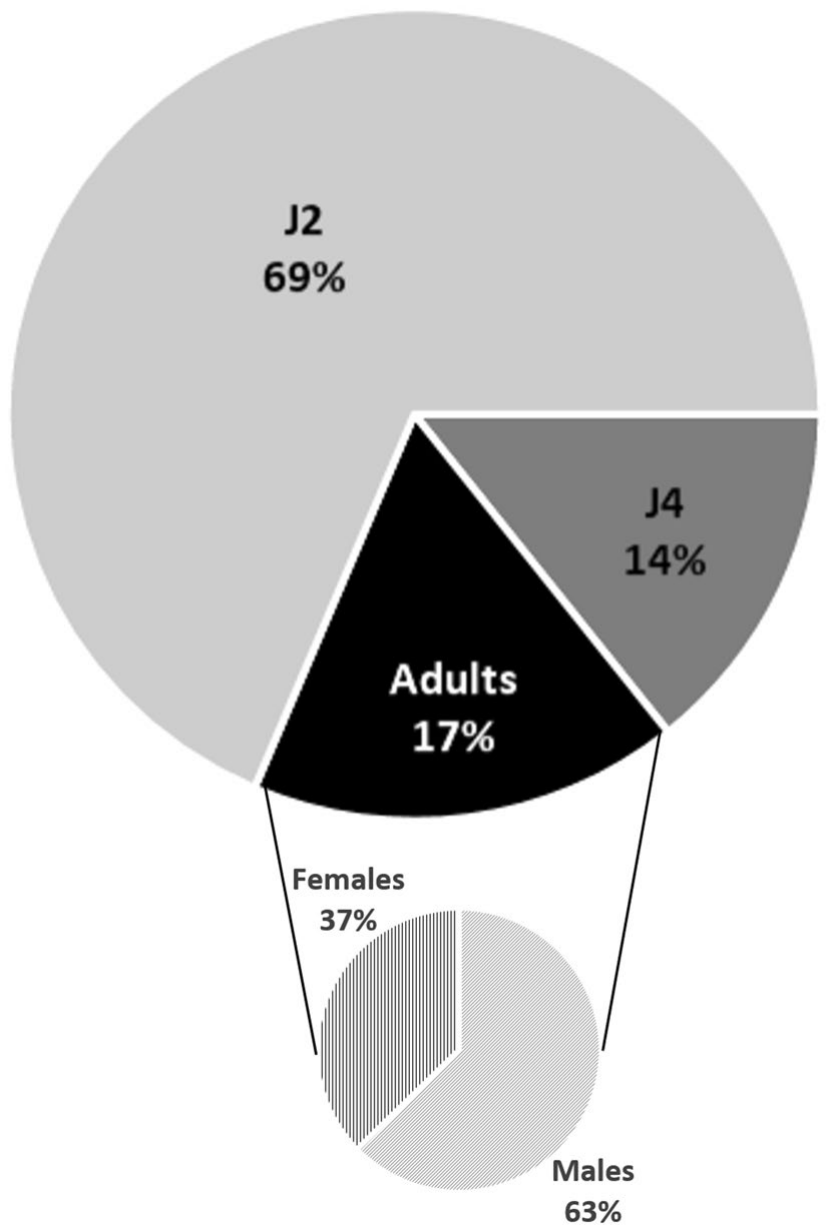
Number of nematodes in different development stages, J_2_, J_4_ and adults (males and females), on *Solanum sisymbriifolium* cv. Sis 6001 root systems. The percentages are averages of the two assays (5 replicates/assay).

The values of GI = 0 and RF = 0 (Table 1 and Supplementary Table S1), and the index 0 in the Bridge & Page (1980) rating chart, obtained for *S. sisymbriifolium* cv. Sis 6001 confirmed its resistance to *M. chitwoodi*, and are consistent with the results obtained by Dias et al. (2012) for other *S. sisymbriifolium* cultivars^17,36^. However, when plants were analyzed separately some variability could be observed between them. Only two replicates (in a total of 10 replicates, 5 per assay) showed the presence of galls, one with one gall and without egg masses and another with 11 galls with 9 egg masses containing 187 eggs in total (Supplementary Table S1). Despite this, there are no statistically significant differences between numbers of galls, egg masses, eggs and RF between *S. linnaeanum* and *S. sisymbriifolium* cv. Sis 6001. Observation of stained roots of *S. sisymbriifolium* cv. Sis 6001 showed some nematodes (total numbers values between 4 and 117 per plant) (Supplementary Table S2). The majority of nematodes inside the roots were J_2_ (69%); of the remainder, 17% were adults, of which 63% were males (Fig. 3).

## Discussion

The choice of the classification system of Sasser et al. (1984) and the rating chart of Bridge & Page (1980) for the resistance studies of *S. linnaeanum* and *S. sisymbriifolium* cv. Sis 6001 to *M. chitwoodi* was because they can be applied to any crop and they also allow results from different studies to be compared without the need to include the susceptible controls^36,37^.

The time that any *Meloidogyne* species needs to complete a generation depends on external factors, such as temperature, humidity, light and the quality and condition of the host plant in relation to its age and nutritional status. All of these factors are crucial as they determine how and when the effects of nematodes on the plant will be most visible^38^. In our laboratory, it was observed that *M. chitwoodi* had a slower rate of development than other *Meloidogyne* isolates. Thus, the plants were uprooted 70 days after inoculation (DAI) instead of the usual 60 DAI. Any differences found between species or replicates can only be attributed to characteristics inherent to the plants as the assay conditions were always the same.

The control of the population density of PPN in the soil is best achieved by resistance in the plant, characterized as the ability of a plant species to inhibit nematode development or reproduction^39^. So far, the capacity of J_2_ penetration into the roots has not been considered as a mechanism for characterization of the resistance. Generally, resistance to nematodes in plants becomes obvious after J_2_ penetration into the roots (i.e. post-infection)^40^. The J_2_ that penetrate the roots of resistant plants may die or leave the roots, but they can also develop to the adult stage, as females without egg production (or the eggs produced are not viable) or as males^41^. Various procedures for determining the resistance of plants to nematodes are used. The most common procedures are the percentage or number of galls or egg masses formed as well as the RF (Pf/Pi) values^37^. The low numbers of nematodes that penetrated the roots of *S. linnaeanum* and *S. sisymbriifolium* cv. Sis 6001 are probably due to substances produced by the roots of these plants, which prevented their penetration. Conceição and collaborators (2012) analyzed the effects of *S. sisymbriifolium* (cvs. Domino, Pion, Sharp and Sis 4004) exudates in five *Meloidogyne* isolates (*M. arenaria*, *M. chitwoodi*, *M. hapla*, *M. hispanica* and *M. javanica*) and showed the complexity and variability of the interactions between RKN and *S. sisymbriifolium*^42^. In this study, the cultivars studied demonstrated low or no inhibitory effect on *M. chitwoodi* hatching. The presence of resistance in some *S. sisymbriifolium* cultivars to PPN, has already been demonstrated for *G. pallida*, *G. rostochiensis*, *M. chitwoodi* and *M. javanica*^17,24^. This was confirmed by Hajihassani et al. (2020) for other major species of RKN (*M. arenaria*, *M. haplanaria* and *M. incognita*), although the effectiveness of resistance to *M. arenaria* varied a lot between *S. sisymbriifolium* cultivars^10^.

In *S. linnaeanum*, in the present work, there was greater nematode penetration of the roots than in *S. sisymbriifolium* cv. Sis 6001, but there was no reproduction in any of the plants, and all nematodes that developed into adults were males. This reaction is a form of active resistance (i.e. post-infection) in which, although the J_2_ can penetrate the roots, they do not find favorable conditions for their normal development and reproduction. Adverse nutrition conditions in younger stages can, by themselves, change the physiology of certain nematodes, eliminating formation of females, egg production or population increase^14^. The larger number of males in the roots may indicate that the plant has a type of resistance that prevents the nematode from feeding normally.

In *S. sisymbriifolium* cv. Sis 6001, although some nematodes penetrated and established in the roots, there was actual reproduction in only one replicate of the first bioassay. The reduced number of nematodes found in the roots may be related to the existence of exudates or compounds produced by the roots of this cultivar that prevent J_2_ penetration^19^. This type of resistance occurs before the nematode penetrates the root (i.e. pre-infection) and is called passive^43^. From analysis of the results of other authors, *S. sisymbriifolium* presents great variability, both between and within cultivars. In some cases, resistant and susceptible plants may co-exist within the same cultivar^17^. In Ali et al. (1992), for *M. incognita*, although *S. sisymbriifolium* was invaded by nematodes which formed some (4–5) swellings (not real galls), the nematodes did not develop beyond J_2_ and there was no egg mass production^21^. Similar results were also reported by Fassuliotis & Bhatt (1982), possibly due to a tracheid discontinuity, due in turn to the formation of a small, thin-walled giant cell. However, for *M. javanica*, using regenerated plants rather than seeds, *S. sisymbriifolium* demonstrated low resistance^44^. Some reports do not mention the *S. sisymbriifolium* cultivar and *Meloidogyne* species used and *S. sisymbriifolium* may react differently to different species of *Meloidogyne*. As reported by Daunay & Dalmasso (1985), *S. sisymbriifolium* was a poor host for *M. arenaria* and *M. incognita* but it was a better host for *M. javanica*^45^. Therefore, caution should be taken when choosing the *S. sisymbriifolium* cultivar to control RKN because the results can change with the cultivar and/or the nematode species considered. The effects of some *S. sisymbriifolium* cultivars, or their plant extracts, on hatching, infectivity and/or reproduction of PCN, RLN and RKN has also been demonstrated by other authors with positive results^17,18,26,27^.

Although *S. sisymbriifolium* is tolerant of cold and heat^21,46^ and is already used as a trap crop in some countries, this plant has already become invasive in some places. However, it is not native to Portugal and is also attacked by pests common to some of our crops^47^. Taking also in consideration, as already mentioned, that *S. sisymbriifolium* cultivars have great intra- and inter-cultivar variability^42,44^ and given the results obtained in this work, *S. linnaeanum* may be a better plant to use against nematodes. It already exists in Portugal and is also a source of resistance to other pests and plant diseases such as Verticillium wilt^33,34^. Despite this, care must also be taken with the *Meloidogyne* species considered and with the plants of *S. linnaeanum* used. Tzortzakakis and collaborators (2006) tested *S. linnaeanum* against *M. incognita* and *M. javanica* and, in both cases, the nematodes reproduced, although in *M. incognita* the numbers of galls and egg masses were significantly lower than in the controls^48^.

The plant species we have considered are resistant, or partially resistant, to some diseases and plant pests. *Solanum linnaeanum* exhibits resistance to Verticillium wilt and Liu and collaborators (2015) succeeded in the transference of this resistance to eggplants through the introgression of the disease resistance gene^34^. In the same way, if the *S. linnaeanum* and *S. sisymbriifolium* resistance genes are isolated, they can be used to confer resistance to plants susceptible to *M. chitwoodi*, such as tomato and potato plants. They have a potential to be used as sources for resistance to *M. chitwoodi* in breeding programs.

In this study, it was demonstrated that *S. linnaeanum* can reduce RKN densities in the soil. Its roots attract *M. chitwoodi* juveniles, removing them from the soil but at the same time reducing the reproduction of the nematodes. The fact that the nematodes developed into males, without females, avoids the risk of reproduction or leaving a dangerous, viable population of nematodes in the soil. Since the number of J_2_ that hatch and penetrate the roots is high, the population density of nematodes in the soil decreases and even those that hatch but do not enter the roots will die. In that way, the use of *S. linnaeanum* as a trap crop, in a crop rotation system, or even as rootstocks, may be an appropriate component of Integrated Pest Management, keeping RKN populations at levels low enough not to cause economic losses, thereby increasing production and crop quality and avoiding or limiting the use of synthetic nematicides. Using a trap crop does not disturb the ecological balance in the soil and the extensive and deep root systems of *S. linnaeanum* grow deeper into the soil than nematicides can penetrate. *Solanum linnaeanum* could be a good alternative to other PPN control measures but more studies are required, including field studies. The crop should be completely removed before flowering or incorporated into the soil as green manure before seeds are produced, preventing it from becoming invasive in areas where it does not yet exist. Also, different conditions should be tested to investigate whether the resistance of the plants is stable at different temperatures. There are no known uses for this plant in Portugal. In this way, it may be possible to find a use for a plant that, despite having existed in Portugal for many years, was only ever considered a weed. In this study, resistance to *M. chitwoodi* in another cultivar of *S. sisymbriifolium* (cv. Sis 6001) was also confirmed.

## Methods

### Nematode isolates

*Meloidogyne chitwoodi* was chosen because it is a problem for potato crops that has already been detected in Portugal and is considered an A2 quarantine pest by EPPO. An isolate of *M. chitwoodi* from the NEMATO-lab of the University of Coimbra, obtained from a potato field in Porto, Portugal^49^, was maintained and multiplied on susceptible tomato plants, *S. lycopersicum* cv. Coração de boi, inoculated with 15 egg masses/plant. The plants were grown in pots filled with 500 g of steam-sterilized soil mix (sand:soil:peat 1:1:1 v/v) and kept in a glasshouse (20–25 °C, 70–75% relative humidity and 12 h photoperiod). Ninety DAI, the plants were uprooted and nematode eggs were extracted with 0.52% sodium hypochlorite (NaOCl) solution, according to Hussey & Barker (1973)^50^. This culture of *M. chitwoodi* is a pure isolate started initially from a single egg mass; its identification was confirmed by esterase phenotype analysis at the beginning and at the end of each assay^51^.

### Plant materials

*Solanum lycopersicum* cv. Coração de boi (tomato), *S. linnaeanum* and *S. sisymbriifolium* cv. Sis 6001 were grown from seeds. Our stock of *S. linnaeanum* is a wild isolate whose seeds were harvested from a plant growing on the roadside in the Algarve. The seeds were germinated at 25–27 °C on moist filter paper in Petri dishes and transplanted singly into 5 cm diameter plastic pots containing 60 cm^3^ of a steam-sterilized mixture of loam soil and sand (1:2 v/v). *Solanum sisymbriifolium* seeds were germinated in a glasshouse in polystyrene plates containing sterile peat. Fifteen days after germination the seedlings of this species were transplanted singly into pots filled with 500 g of steam-sterilized soil mix (sand:soil:peat 1:1:1 v/v). The potato plants, *S. tuberosum* ssp. *tuberosum* L. (cv. Désirée), were obtained from pieces of potato tubers with sprouts in pots with the same mixture of soil (sand:soil:peat 1:1:1 v/v). All plants were kept in a glasshouse under the same conditions as described above.

### Pathogenicity tests

Five four-weeks-old plants from each species were inoculated with 5000 M*. chitwoodi* eggs (initial population density, Pi). To confirm the soil sterility one plant of each species was potted in the sterilized soil mix and not inoculated. Five susceptible tomato plants cv. Coração de boi and five susceptible potato plants cv. Désirée were also inoculated to confirm the viability of the inocula. Pots were kept in the conditions already mentioned and the plants were watered daily. Seventy DAI the plants were uprooted and the root systems washed. The root systems were stained with phloxine B (0.0015% solution) for 15 min^52^, and galls and eggs masses were counted. Eggs were extracted as described above and counted to determine the final population (Pf). Resistance rating of the species were based on Gall Index (GI) and Reproduction Factor (RF = Pf / Pi), according to the modified quantitative scheme of Canto-Sáenz (1985)^41,53^. The evaluation of the degree of resistance of the plants was based on GI and RF^37^.

Roots that had less than 100 egg masses were stained with acid fuchsin^54^, and the numbers of the different developmental stages of *M. chitwoodi* were recorded. Resistance ratings of these plants were based on the RKN rating chart of Bridge & Page (1980)^36^. This scale is based on the percentage and types of roots galled from 0 (0%, no galls) to 10 (100% galled). The roots were observed using a routine stereo microscope Leica M80, at a magnification of 60 x, and the nematodes found were transferred onto a glass slide and observed with an optical microscope Leica DM2500, at a magnification of 400 ×. The identification of the different developmental stages was done by comparing their morphological characteristics with those described for *M. incognita* (Supplementary Fig. S2)^41,55^.

The assay was done twice, using the same conditions each time.

### Statistical analysis

The data (values obtained for galls, egg masses and eggs counts and RF), were confirmed to meet the statistical assumptions of normality and homogeneity of variances (one way ANOVA), and were submitted to analysis of variance and the means compared by LSD (P < 0.05) using Statistic 10 software (Statsoft Inc.).

## Supplementary information

Supplementary Information 1.

Supplementary Information 2.

Supplementary Information 3.
