# Supplementary figures and images for: Solanum linnaeanum and Solanum sisymbriifolium as a sustainable strategy for the management of Meloidogyne chitwoodi
Source: Sci Rep. 2021 Feb 10;11:3484. doi: 10.1038/s41598-020-77905-2 (PMC7875996; doi:10.1038/s41598-020-77905-2)

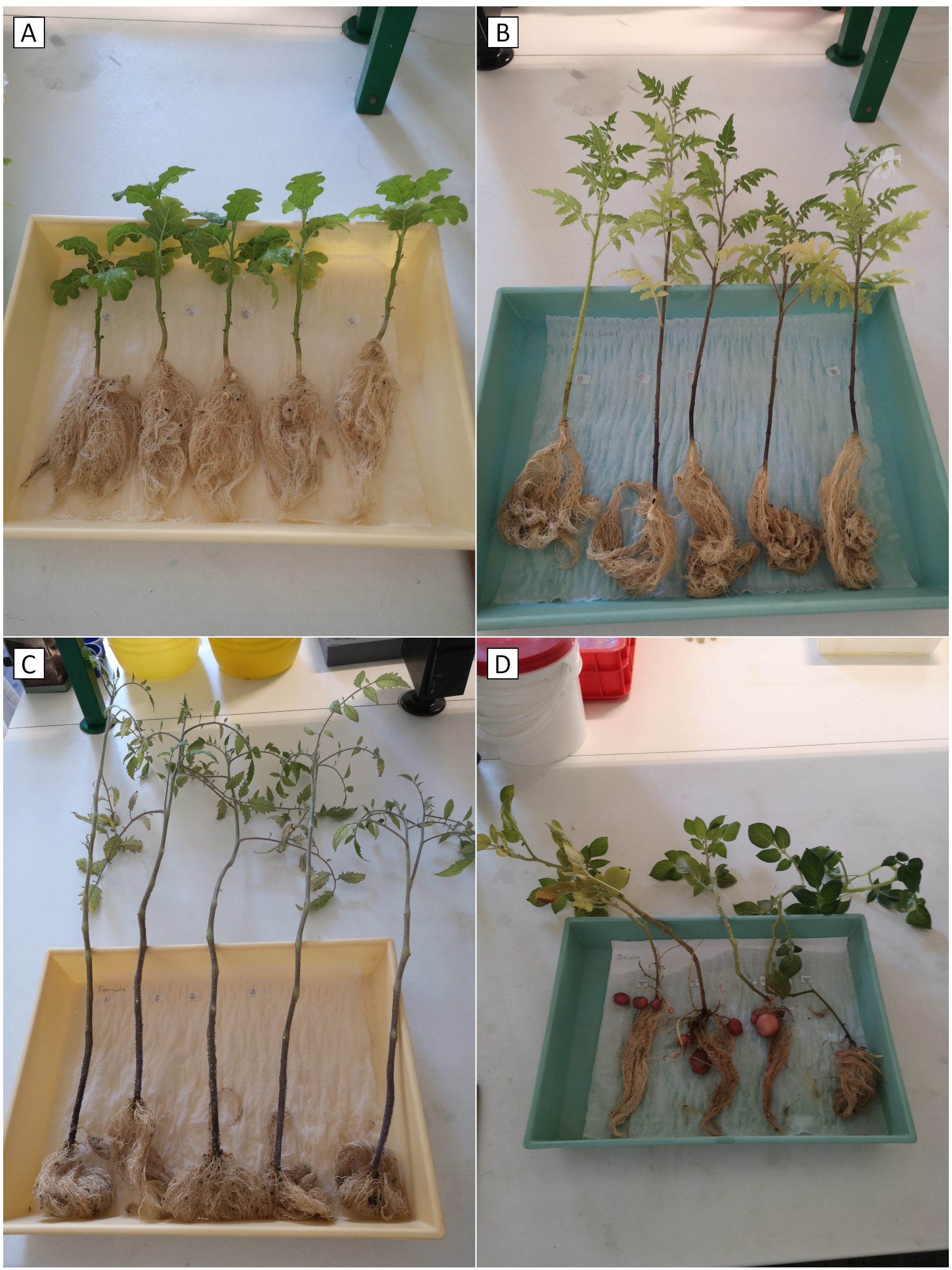

Supplement: Supplementary file 1 — Supplementary Information 1. [file 41598_2020_77905_MOESM1_ESM.jpg]

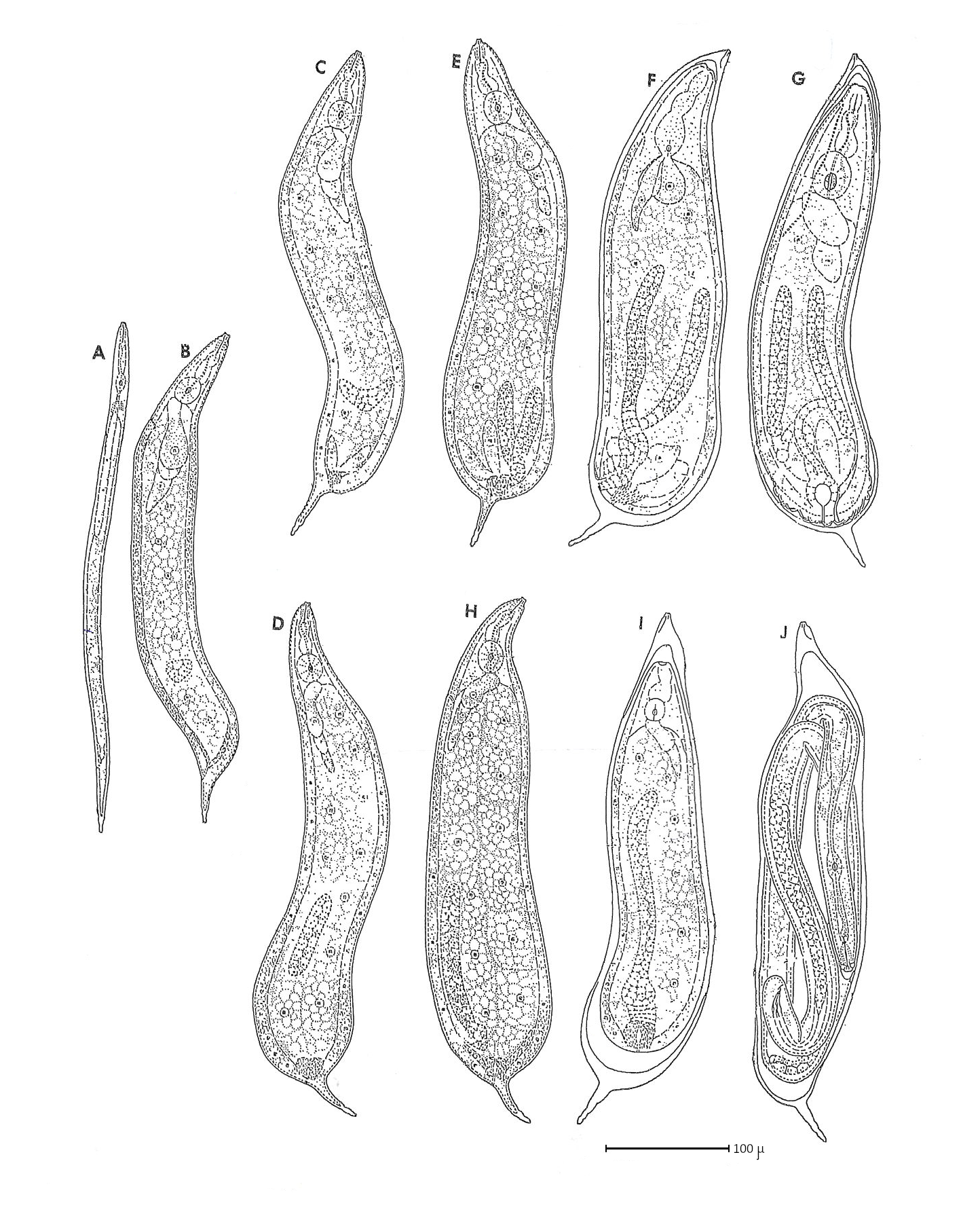

Supplement: Supplementary file 2 — Supplementary Information 2. [file 41598_2020_77905_MOESM2_ESM.png]
