# Supplementary material for: Solanum linnaeanum and Solanum sisymbriifolium as a sustainable strategy for the management of Meloidogyne chitwoodi
Source: Sci Rep. 2021 Feb 10;11:3484. doi: 10.1038/s41598-020-77905-2 (PMC7875996; doi:10.1038/s41598-020-77905-2)
Supplement: Supplementary file 3 — Supplementary Information 3. [file 41598_2020_77905_MOESM3_ESM.docx]

*Solanum linnaeanum* and *Solanum sisymbriifolium* as a sustainable strategy for the management of *Meloidogyne chitwoodi*

Laura Soraia Perpétuo^1,2,3*^, Maria J. M. da Cunha^1,2^, Maria Teresa Batista^3^ & Isabel Luci Conceição^1^

^1^Centre for Functional Ecology - Science for People & the Planet (CFE), Department of Life Sciences, University of Coimbra, P-3000 456 Coimbra, Portugal

^2^Polytechnic Institute of Coimbra, High School of Agriculture, Department of Agronomic Sciences and Research Centre for Natural Resources, Environment and Society (CERNAS), Bencanta, P-3045 601 Coimbra, Portugal

^3^Chemical Process Engineering and Forest Products Research Centre (CIEPQPF), Faculty of Pharmacy of the University of Coimbra, P-3000 548 Coimbra, Portugal

^*^Corresponding author: soraiaperpetuo@gmail.com

**Supplementary Figure S1.** Solanum linnaeanum (A), S. sisymbriifolium cv. Sis 6001 (B), S. lycopersicum cv. Coração de boi (C) and S. tuberosum ssp. tuberosum cv. Désirée (D) plants.

**Supplementary Figure S2.** Development of Meloidogyne incognita. A – Infective second-stage juvenile (J_2_); B – Sexually undifferentiated J_2_; C – J_2_ female; D - J_2_ male; E – Late J_2_ female; F – Fourth-stage juvenile (J_4_) female; G - Adult female shortly after fourth stage; H - Late J_2_ male; I - J_4_ male; J - Adult male shortly after fourth stage (Adapted from Triantaphyllou & Hirschmann, 1960 in Taylor & Sasser, 1978 (41, 55)).

| ***Solanum* species and cultivars** | **Assay** | **Replicate** | **G** | **EM** | **P_i_** | **P_f_** | **RF** | **GI** | **RD** |
| --- | --- | --- | --- | --- | --- | --- | --- | --- | --- |
| ***S. linnaeanum*** | **1** | 1 | 0 | 0 | 5000 | 0 | 0 | 0 | R |
|  |  | 2 | 0 | 0 | 5000 | 0 | 0 | 0 | R |
|  |  | 3 | 0 | 0 | 5000 | 0 | 0 | 0 | R |
|  |  | 4 | 0 | 0 | 5000 | 0 | 0 | 0 | R |
|  |  | 5 | 0 | 0 | 5000 | 0 | 0 | 0 | R |
|  | **2** | 6 | 0 | 0 | 5000 | 0 | 0 | 0 | R |
|  |  | 7 | 0 | 0 | 5000 | 0 | 0 | 0 | R |
|  |  | 8 | 0 | 0 | 5000 | 0 | 0 | 0 | R |
|  |  | 9 | 0 | 0 | 5000 | 0 | 0 | 0 | R |
|  |  | 10 | 0 | 0 | 5000 | 0 | 0 | 0 | R |
| ***S. sisymbriifolium* cv. Sis 6001** | **1** | 1 | 11 | 9 | 5000 | 187 | 0 | 3 | HS |
|  |  | 2 | 1 | 0 | 5000 | 0 | 0 | 1 | R |
|  |  | 3 | 0 | 0 | 5000 | 0 | 0 | 0 | R |
|  |  | 4 | 0 | 0 | 5000 | 0 | 0 | 0 | R |
|  |  | 5 | 0 | 0 | 5000 | 0 | 0 | 0 | R |
|  | **2** | 6 | 0 | 0 | 5000 | 0 | 0 | 0 | R |
|  |  | 7 | 0 | 0 | 5000 | 0 | 0 | 0 | R |
|  |  | 8 | 0 | 0 | 5000 | 0 | 0 | 0 | R |
|  |  | 9 | 0 | 0 | 5000 | 0 | 0 | 0 | R |
|  |  | 10 | 0 | 0 | 5000 | 0 | 0 | 0 | R |
| ***S. lycopersicum* cv. Coração de boi** | **1** | 1 | > 100 | > 100 | 5000 | 37547 | 8 | 5 | S |
|  |  | 2 | > 100 | > 100 | 5000 | 63867 | 13 | 5 | S |
|  |  | 3 | > 100 | > 100 | 5000 | 66453 | 13 | 5 | S |
|  |  | 4 | > 100 | > 100 | 5000 | 41173 | 8 | 5 | S |
|  |  | 5 | > 100 | > 100 | 5000 | 114133 | 23 | 5 | S |
|  | **2** | 6 | > 100 | > 100 | 5000 | 17813 | 4 | 5 | S |
|  |  | 7 | > 100 | > 100 | 5000 | 20053 | 4 | 5 | S |
|  |  | 8 | > 100 | > 100 | 5000 | 27307 | 5 | 5 | S |
|  |  | 9 | > 100 | > 100 | 5000 | 30187 | 5 | 5 | S |
|  |  | 10 | > 100 | > 100 | 5000 | 28800 | 6 | 5 | S |
| ***S. tuberosum* ssp. *tuberosum* cv. Désirée** | **1** | 1 | > 100 | 63 | 5000 | 9680 | 2 | 5 | S |
|  |  | 2 | > 100 | > 100 | 5000 | 211947 | 42 | 5 | S |
|  |  | 3 | > 100 | > 100 | 5000 | 43627 | 9 | 5 | S |
|  |  | 4 | > 100 | 55 | 5000 | 4347 | 1 | 5 | HS |
|  |  | 5 | The plant died before the end of the assay | | | | | | |
|  | **2** | 6 | > 100 | > 100 | 5000 | 21973 | 4 | 5 | S |
|  |  | 7 | 89 | > 100 | 5000 | 13973 | 3 | 4 | S |
|  |  | 8 | > 100 | > 100 | 5000 | 42667 | 9 | 5 | S |
|  |  | 9 | > 100 | > 100 | 5000 | 46933 | 9 | 5 | S |
|  |  | 10 | > 100 | > 100 | 5000 | 23467 | 5 | 5 | S |

**Supplementary Table S1.** Numbers of galls (G) and egg masses (EM), initial population (P_i_), final population (P_f_) and reproduction factor (RF) of Meloidogyne chitwoodi in Solanum linnaeanum, S. sisymbriifolium cv. Sis 6001, S. lycopersicum cv. Coração de boi and S. tuberosum ssp. tuberosum cv. Désirée, 70 days after inoculation with 5000 eggs/plant, together with respective gall indices (GI) and resistance degree (RD: HS = hypersusceptible; R = resistant; S = susceptible).

| ***Solanum* species and cultivars** | **Assay** | **Replicate** | **Infective J_2_** | **Undifferentiated J_2_** | **Males** | | | **Females** | | |
| --- | --- | --- | --- | --- | --- | --- | --- | --- | --- | --- |
|  |  |  |  |  | **J_2_** | **J_4_** | **Adults** | **J_2_** | **J_4_** | **Adults** |
| ***S. linnaeanum*** | **1** | 1 | 45 | 390 | 11 | 14 | 94 | 0 | 0 | 0 |
|  |  | 2 | 14 | 417 | 13 | 0 | 146 | 0 | 0 | 0 |
|  |  | 3 | 21 | 528 | 17 | 0 | 253 | 0 | 0 | 0 |
|  |  | 4 | 9 | 413 | 36 | 0 | 300 | 0 | 0 | 0 |
|  |  | 5 | 7 | 352 | 22 | 0 | 502 | 0 | 0 | 0 |
|  | **2** | 6 | 6 | 114 | 54 | 102 | 282 | 3 | 0 | 0 |
|  |  | 7 | 5 | 27 | 50 | 78 | 169 | 0 | 0 | 0 |
|  |  | 8 | 4 | 10 | 68 | 108 | 379 | 0 | 0 | 0 |
|  |  | 9 | 1 | 6 | 47 | 235 | 322 | 0 | 0 | 0 |
|  |  | 10 | 1 | 13 | 48 | 184 | 340 | 0 | 0 | 0 |
| ***S. sisymbriifolium* cv. Sis 6001** | **1** | 1 | 6 | 38 | 0 | 0 | 1 | 9 | 43 | 20 |
|  |  | 2 | 0 | 3 | 0 | 0 | 0 | 0 | 0 | 1 |
|  |  | 3 | 15 | 24 | 2 | 0 | 10 | 1 | 0 | 0 |
|  |  | 4 | 2 | 3 | 0 | 0 | 3 | 0 | 0 | 1 |
|  |  | 5 | 12 | 7 | 0 | 0 | 0 | 0 | 0 | 0 |
|  | **2** | 6 | 23 | 1 | 0 | 0 | 0 | 0 | 0 | 0 |
|  |  | 7 | 12 | 4 | 0 | 0 | 0 | 0 | 0 | 0 |
|  |  | 8 | 25 | 23 | 5 | 4 | 23 | 0 | 0 | 0 |
|  |  | 9 | 10 | 3 | 0 | 0 | 0 | 0 | 0 | 0 |
|  |  | 10 | 12 | 1 | 0 | 0 | 0 | 0 | 0 | 0 |

**Supplementary Table S2.** Developmental stages of Meloidogyne chitwoodi in Solanum linnaeanum and S. sisymbriifolium cv. Sis 6001, by comparing their morphological characteristics with those described for M. incognita (41, 55) and numbers of nematodes/stage, 70 days after inoculation with 5000 eggs/plant.
